# Dr. D. W. Yandell’s Operations for Stone in the Bladder

**Published:** 1853-07

**Authors:** 


### DR. D. W. YANDELL'S OPERATIONS FOR STONE IN THE BLADDER.

Dr. D. W. Yandell has obligingly furnished us with the following brief notes of his operations for stone in the bladder:

"*Case* 1.—William Allen, of Carroll county, Tennessee, aet. 20; the subject of urinary calculus since his third year. Operated May, 1850, in presence of Drs. Wright and Bell, and several other professional gentlemen, of Huntingdon. The calculus was of a flattened, oval shape, soft, exceedingly friable, and adherent, by its edges and one side, to the *bas fond* of the bladder. It weighed between three and four ounces, and measured four inches in circumference Recovery slow, but steady and complete. There has been no return of the disease.

*Case* 2.—A little son of Mr. B., in the same county. three years and a half old, had suffered with symptoms of stone since soon after his birth.

Assisted by the same gentlemen, and by Dr. Wilson W. Yandell, of Gibson county, I operated upon him a few days after performing my first operation, and removed a stone of the size of a pigeon's egg. The wound healed by the first intention; in twenty-four hours the urine passed by the urethra. The calculus was soft and friable, as in the first case.

*Case* 3.—The youngest son of Hon. William Fitzgerald, near Paris, Tenn., between three and four years of age, had been troubled with symptoms of vesical calculus since his early infancy. In June, 1850, with the assistance of Drs. Taliaferro and Warren, of Paris, and Dr. Wright, of Huntingdon, I extracted from the bladder of the little patient two smooth, white, soft calculi, each about the size of a partridge's egg. The recovery was rapid, and his general health has become perfect. In neither of these cases have there been any indications of the formation of other calculi.

*Case* 4.—The subject of the following observation is the son of Robert Douglass, Esq., of Sumner county, Tennessee. When the operation was performed, in December, 1850, the little boy was in his fourth year, and robust, though not large of his age. My friend Dr. Elmcre Douglass, of Gallatin, a relative of the patient, and Drs. Duval and Foster were present at the operation, and afforded me the requisite assistance. The stone was small, rough, and very soft. The patient had a good recovery, and has enjoyed uninterrupted health up to the present time.

*Case* 5—The subject in this case was a little boy, the son of Mr. Shaffner, of Carroll county, Tenn. The operation was performed with ease, and for two days everything promised well. Forty-eight hours after it was done the wound had almost closed, and the urine had begun to be voided by the natural passage. Soon after this, the patient was seized with a chill, which persisted for thirty six hours. On rallying from the chill—which, for some time, I was almost certain he would never do—the reaction was excessive, and the end of all was phlebitis, commencing at the wound and extending to the tip of the great toe of the left foot. The veins in both groins were the seat of intense inflammation. It was not until the tenth day that I despaired of his recovery The constitution of the little patient was enfeebled and poor, and to this, I suppose, may be ascribed the occurrence of the phlebitis.

In each of these operations chloroform was used, and the patients rendered unconscious of the pain. The instrument employed was the bistoury, and the method pursued that of Mr. Liston."

June 24th, 1853.

